# Engineering ligand chemistry on Au_25_ nanoclusters: from unique ligand addition to precisely controllable ligand exchange†

**DOI:** 10.1039/d3sc01177a

**Published:** 2023-06-22

**Authors:** Jiangtao Zhao, Abolfazl Ziarati, Arnulf Rosspeintner, Yanan Wang, Thomas Bürgi

**Affiliations:** a Department of Physical Chemistry, University of Geneva 30 Quai Ernest-Ansermet 1211 Geneva 4 Switzerland abolfazl.ziarati@unige.ch thomas.buergi@unige.ch; b Department of Chemical Engineering, University of Michigan Ann Arbor 2800 MI USA

## Abstract

Au_25_ nanoclusters (NCs) protected by 18 thiol-ligands (Au_25_SR_18_, SR is a thiolate ligand) are the prototype of atomically precise thiolate-protected gold NCs. Studies concerning the alteration of the number of surface ligands for a given Au_25_SR_18_ NC are scarce. Herein we report the conversion of hydrophobic Au_25_PET_18_ (PET = 2-phenylethylthiolate) NCs to Au_25_SR_19_ [Au_25_PET_18_(metal complex)_1_] induced by ligand exchange reactions (LERs) with thiolated terpyridine-metal complexes (metal complex, metal = Ru, Fe, Co, Ni) under mild conditions (room temperature and low amounts of incoming ligands). Interestingly, we found that the ligand addition reaction on Au_25_PET_18_ NCs is metal dependent. Ru and Co complexes preferentially lead to the formation of Au_25_SR_19_ whereas Fe and Ni complexes favor ligand exchange reactions. High-resolution electrospray ionization mass spectrometry (HRESI-MS) was used to determine the molecular formula of Au_25_SR_19_ NCs. The photophysical properties of Au_25_PET_18_(Ru complex)_1_ are distinctly different from Au_25_PET_18_. The absorption spectrum is drastically changed upon addition of the extra ligand and the photoluminescence quantum yield of Au_25_PET_18_(Ru complex)_1_ is 14 times and 3 times higher than that of pristine Au_25_PET_18_ and Au_25_PET_17_(Ru complex)_1_, respectively. Interestingly, only one surface ligand (PET) could be substituted by the metal complex when neutral Au_25_PET_18_ was used for ligand exchange whereas two ligands could be exchanged when starting with negatively charged Au_25_PET_18_. This charge dependence provides a strategy to precisely control the number of exchanged ligands at the surface of NCs.

## Abstract 2

The precisely controllable ligand exchange and ligand addition reaction on Au_25_SR_18_ are achieved *via* the manipulation of incoming ligands and charge of Au_25_; this provides a novel tool to control the surface ligand chemistry on Au nanoclusters.

## Graphical abstract

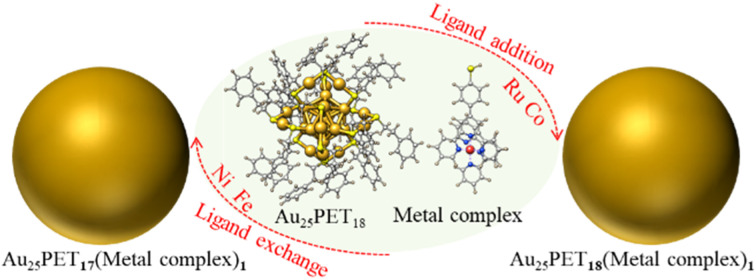

## Introduction

Thiolate-protected metal nanoclusters (NCs) have attracted tremendous attention in the past few decades,^1–4^ and triggered extensive research interests in many emerging fields, such as biological applications,^5,6^ electro(photo)catalysis for energy conversion^7,8^ and sensing,^9,10^ owing to well-defined structures, unique optical and molecular-like properties.^11^ To date, dozens of different thiolate-protected gold NCs are known (denoted as Au_*m*_SR_*n*_, *m* and *n* indicate the number of gold atoms and thiolate ligands, respectively), such as Au_25_SR_18_, Au_38_SR_24_, Au_102_SR_44_, Au_144_SR_60_, *etc*.^12^ However, to further extend the range of possible applications of NCs, the research on diversification of Au NC species with modified morphologies and properties remains important.

Thiolate-ligands on the surface of the Au NCs play an important role during the synthesis of NCs,^13^ and influence the physicochemical properties of NCs, boost their functionalities and affect the NC size.^14–16^ The number of surface ligands for a given Au nuclearity is typically well-defined. For example, Au_25_SR_18_, the prototypical thiolate-protected gold NC contains exactly 18 ligands (the Au_13_ core is surrounded by 6 Au_2_SR_3_ staples). This NC seems the ideal candidate to study the derivatization chemistry of Au NCs. Nowadays, Au NCs are usually fabricated by a well-recognized synthetic method using neutral or anionic thiolate ligands, such as 2-phenylethanethiol (PET), glutathione, mercaptocarboxylic acids and so on.^17–19^ The direct synthesis of cationic-ligand-protected Au NCs is limited because of coulombic attractions between the anionic Au ion (AuCl_4_^−^) and the cationic thiol and coulombic repulsions caused by abundant cationic ligands at the surface of Au NCs.^20,21^ Ligand exchange reactions (LERs), in which surface ligands on Au NCs are substituted with new thiolate ligands, thus functionalizing the NCs, provide a crucial post-synthesis methodology for surface chemical modification of Au NCs.^22,23^ Particularly, chromophore-functionalized Au NCs were prepared and their photophysical properties studied.^24–27^ For instance, the optical behavior was investigated in porphyrin functionalized chiral Au_38_SR_24_ NC,^28^ and a directional electron transfer from the Au_25_ NC to the pyrene was studied in pyrene-coupled Au_25_ NCs.^29^ In addition, the ligand-exchange position controlled by steric hindrance of incoming ligands was revealed in porphyrinthiol-substituted Au_25_ NCs system.^30^ Ligand exchange with free ligand can be divided into two categories: (i) part or all surface ligands are replaced by the new ligands without changing the size and structure of the original Au NCs;^31^ (ii) ligand exchange induces a size/structure transformation, which was reported by Jin and co-workers.^32^ It provided an easy way to explore the diversity of NCs and generate new NCs with different composition and properties and to study the transformation mechanisms,^33^ for example, from Au_25_SR_18_ to Au_28_SR'_20_,^34^ from Au_144_SR_60_ to Au_133_SR'_52_, and so on.^35^ Some of us previously reported the transformation from Au_25_SR_18_ to Au_28_SR_21_ induced by LER with a chiral ligand under mild conditions in organic phase.^36^ Xie and co-workers recently discovered a new hydrophilic Au_25_SR_19_ NC by adding excess thiolate ligands (MHA = 6-mercaptohexanoic acid) to Au_25_MHA_18_ NCs,^37^ and the transformation mechanism was studied and proposed to occur through an oxidative etching process. In that work, the additional ligand was identical to the one protecting the original NC and the ligand addition process involving different ligands was not investigated.

In this work, we successfully obtained hydrophobic Au_25_SR_19_ by precisely adding one metal complex as ligand on the Au_25_PET_18_ surface. We demonstrated that the single ligand addition on Au_25_PET_18_ is metal dependent (metal = Ru, Fe, Co, Ni), so that the ligand addition reaction can be precisely controlled. Time-resolved high resolution ESI and UV-vis spectra were recorded to monitor the transformation process of Au_25_SR_19_, and a structure of the Au_25_SR_19_ NC is also proposed. Furthermore, it is worth to mention that neither excess thiolate ligand nor high temperature was needed to trigger this transformation. More interestingly, the number of surface ligands that can be replaced by metal complexes during LER depended on the charge of parent Au NCs.

## Results and discussions

**Fig. 1:**
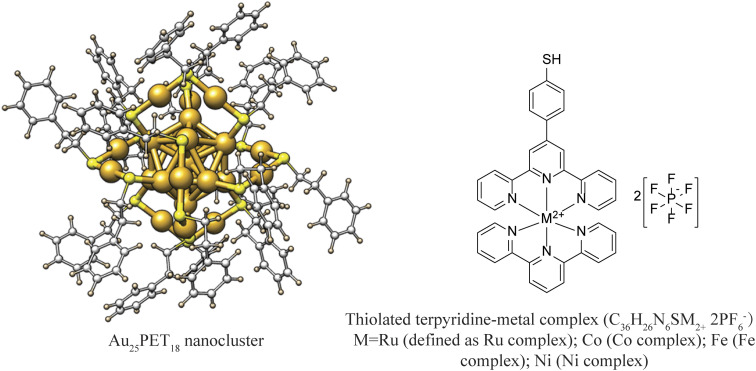
Schematic diagram of Au_25_PET_18_ NC and thiolated terpyridine-metal complex (counter ions: 2PF_6_^−^).

**Fig. 2:**
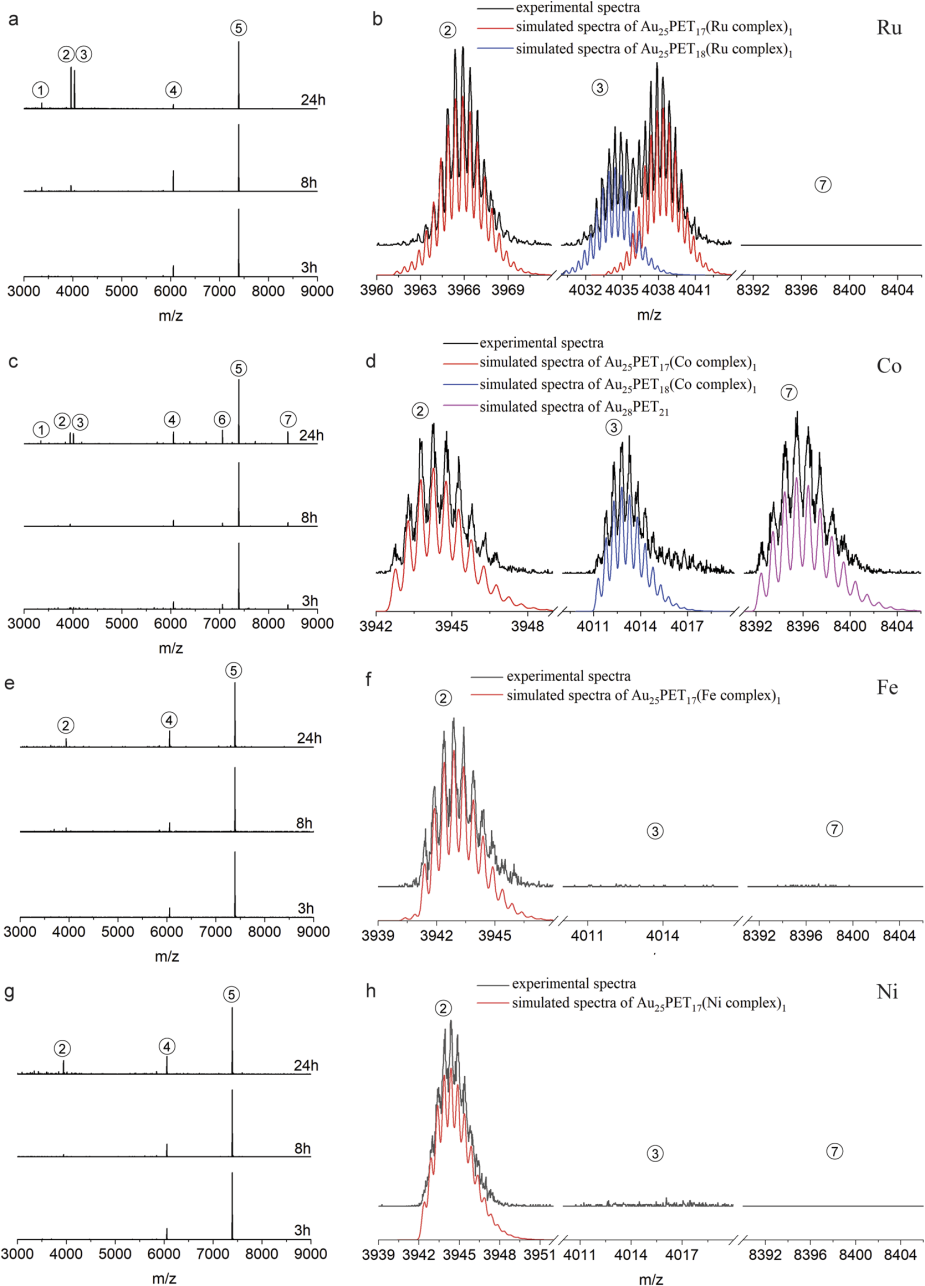
Time-dependent ESI-MS during LER with Ru complex (a and b), Co complex (c and d), Fe complex (e and f) and Ni complex (g and h) at NCs/complex ratio 1 : 2. (b, d, f and h) Show the comparison of experimental and simulated isotopic patterns of different species after reaction for 24 h (enlarged from a, c, e and g, respectively). (Isotopic patterns distance = 0.5 in peak 1–3 indicated the charge *z* = 2+, distance = 1 in peak 4–7 showed the charge *z* = 1+; the spectra are normalized with respect to peak 5).

Neutral Au_25_PET_18_ (PET = 2-phenylethanethiol) was fabricated and purified according to previous reports and used as model NC in the following experiments.^38^ UV-vis absorption spectra (ESI, Fig. S1a†) and high-resolution electrospray ionization mass spectra (HRESI-MS) (ESI, Fig. S1b†) of as-prepared neutral Au_25_PET_18_ NC indicates successful synthesis and high purity.^38,39^ ESI, Fig. S2† shows ^1^H-NMR spectra of the Ru complex. Characteristic absorption spectra and ESI-MS of different metal complexes are displayed in ESI, Fig. S3 and Table. S1,† respectively. LER was performed by mixing Au_25_PET_18_ in dichloromethane and metal complex in acetonitrile (Fig. 1) in a glovebox at room temperature at NC/complex ratios of 1 : 2 and 1 : 4, respectively. The progress of the reaction was monitored by HRESI-MS (Fig. 2) at NC/complex ratio 1 : 2. Peak 5 at *m*/*z* = 7394.2 and peak 4 located at *m*/*z* = 6058 are assigned to the unreacted Au_25_PET_18_ and its prominent fragment.

Fig. 2a shows the time-dependent ESI-MS during LER with the Ru complex at a ratio of 1 : 2, both experimental and simulated spectra of the emerging products are shown in Fig. 2b. As demonstrated in Fig. 2a and b, peak 2 and peak 3 are associated with doubly charged species because the interval between isotopic peaks is 0.5 *m*/*z*. Peak 2 at *m*/*z* = 3965.9 indicates that one PET ligand was substituted by the Ru complex, [Au_25_PET_17_(Ru complex)_1_]^2+^. The isotopic pattern of peak 3 from *m*/*z* = 4030 to *m*/*z* = 4044 demonstrates that two species overlapped in this region, the right part at *m*/*z* = 4038.3 is also consistent with one ligand exchange species, but containing one counterion PF_6_^−^. The left part located at *m*/*z* = 4034.5 is assigned to a species containing one incoming ligand (Ru complex) added to Au_25_PET_18_, [Au_25_PET_18_(Ru complex)_1_]^2+^, a species with the same nuclearity but different ligand number. Noteworthily, peak 1 at *m*/*z* = 3366.1 represents the fragment from Au_25_PET_18_(Ru complex)_1_, after losing Au_4_PET_4_. Such fragmentation is a common phenomenon for Au NCs during MS analysis.^36^

Fig. 2c and d show the time-dependent HRESI-MS for the reaction with Co complex including detail views of some selected peaks. Peak 2 at *m*/*z* = 3944.8 in Fig. 2c and its isotopic pattern in Fig. 2d indicates a NC where one surface ligand was replaced by a Co complex, [Au_25_PET_17_(Co complex)_1_]^2+^. In addition, comparison of the experimental isotope pattern of peak 3 at *m*/*z* = 4013.4 and the simulated curve of Au_25_PET_18_(Co complex)_1_ in Fig. 2d, also confirmed ligand addition. Peak 1 at *m*/*z* = 3344.8 is a fragment from the Au_25_PET_18_ (Co complex)_1_ NC after loss of Au_4_PET_4_. More interestingly, the perfect matching of simulated isotope pattern and the experimental curve for the species at *m*/*z* = 8396.8 indicates the formation of Au_28_PET_21_ (peak 7 in Fig. 2c and d). Peak 6 at *m*/*z* = 7059.4 could be due to the fragment from unreacted Au_25_PET_18_ after loss of Au_1_PET_1_ and Au_28_PET_21_ after loss of Au_4_PET_4_. ESI-MS results after reaction for 3 h, 8 h and 24 h with Fe complex and Ni complex are shown in Fig. 2e, f, g and h, respectively. Both peak 2 in Fig. 2e and f at *m*/*z* = 3943.2 and peak 2 in Fig. 2g and h at *m*/*z* = 3944.7 correspond to ligand substitution with Fe complex and Ni complex, [Au_25_PET_17_(Fe complex)_1_ and Au_25_PET_17_(Ni complex)_1_], respectively. Thus, the ligand-exchange species is the dominant product when reacting Au_25_PET_18_ with Fe and Ni complex at a ratio of 1 : 2. Neither ligand addition nor size transformation was observed.

Table 1 summarizes the metal-dependent products after LER for 24 h at a NC/complex ratio of 1 : 2. It clearly shows that Ru and Co complexes can induce both ligand exchange and ligand addition on Au_25_PET_18_, and the transformation from Au_25_ to Au_28_ also appeared in the case of the Co complex. Nevertheless, only ligand-exchange products were observed when reacting with Fe and Ni complexes.

**Table 1:** Summary of metal-dependent products after LER for 24 h at NC/complex ratio 1 : 2. (✓ means the reaction can take place, ✗ indicates that the reaction was not observed)

	Ru	Co	Fe	Ni
Ligand exchange reaction	✓	✓	✓	✓
Ligand addition reaction	✓	✓	✗	✗
Au_28_PET_21_	✗	✓	✗	✗

**Fig. 3:**
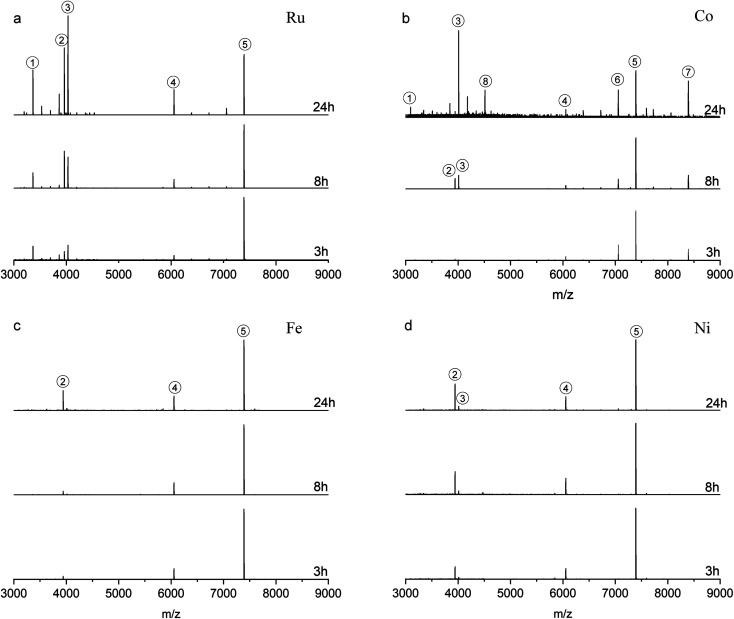
ESI-MS after reaction with Ru complex (a), Co complex (b), Fe complex (c), Ni complex (d) at NCs/complex ratio 1 : 4 (the spectra are normalized with respect to peak 5).

The reactions were also studied at a 1 : 4 NC: complex ratio (Fig. 3). Compared with the reactions at 1 : 2 ratio, Fig. 3a (reaction with Ru complex) and Fig. 3b (reaction with Co complex) show that a higher amount of metal complex is more favorable to induce the ligand addition on Au_25_PET_18_, forming Au_25_SR_19_. Noticeably, peak 3 in Fig. 3a and b after 24 h reaction, located at *m*/*z* = 4034.5 and *m*/*z* = 4013.4, are still assigned to Au_25_SR_19_, [Au_25_PET_18_(Ru complex)_1_]^2+^ and [Au_25_PET_18_(Co complex)_1_]^2+^, respectively. Moreover, peak 8 in Fig. 3b appearing at *m*/*z* = 4014.6 (*z* = 2+) indicates a new NC species corresponding to the addition of one Co complex to Au_28_PET_21_, [Au_28_PET_21_(Co complex)_1_]^2+^, also demonstrating the ligand addition reaction for another NC. Concerning LER with Fe and Ni complex at 1 : 4 ratio, ligand-exchange species Au_25_PET_17_(Metal complex)_1_ is still the main product (shown in Fig. 3c and d). Peak 3 in Fig. 3d at *m*/*z* = 4013.3 confirmed that a small amount of Au_25_SR_19_, [Au_25_PET_18_(Ni complex)_1_]^2+^, formed during the reaction with Ni complex at high ratio.

Since the Ru complex can induce both ligand exchange and ligand addition reactions, it was selected to further investigate the ligand addition process during LER at a 1 : 10 NC: complex ratio. HRESI-MS (ESI, Fig. S4†) indicates that most of the initial Au_25_PET_18_ was converted to Au_25_SR_19_ (peak 1 at 3366.1 and peak 3 at 4034.5) after 24 h, demonstrating that excess ligand is beneficial for the formation of Au_25_SR_19_, and the addition of a second complex was not observed.

In order to make sure that the ESI-MS signal of Au_25_SR_19_ is not due to non-covalent interaction (π–π stacking) between the surface ligand of Au_25_PET_18_ and was prepared and mixed with Au_25_PET_18_. After reaction for 24 h, the sample was analyzed by HRESI-MS (ESI, Fig. S5†). The ESI mass spectra do not show the peak at *m*/*z* = 3959.8 which is assigned to the ligand addition. This indicates that the ligand is added through covalent bonding and not through π–π interaction.

Table 2 summarizes the proposed species and their corresponding *m*/*z* peaks based on the results in Fig. 2 and 3. Ru and Co complexes favor ligand addition (peak 3) reaction. The Co complex can also induce the transformation from Au_25_ to Au_28_ (peak 7) during LER. Nevertheless, with Fe and Ni complexes, ligand exchange (peak 2) is the preferred reaction.

**Table 2:** Proposed species and its corresponding ESI-MS peaks. S atoms are presented in yellow, *z* means charge

Species	*m*/*z*
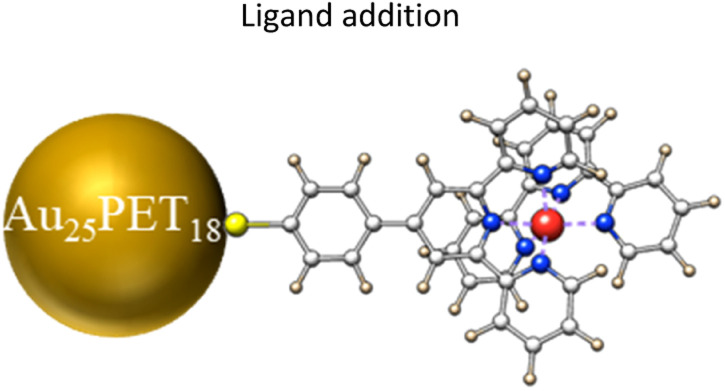	(*z* = 2+), M = Ru: 4034.5, M = Co: 4013.4, peak 3
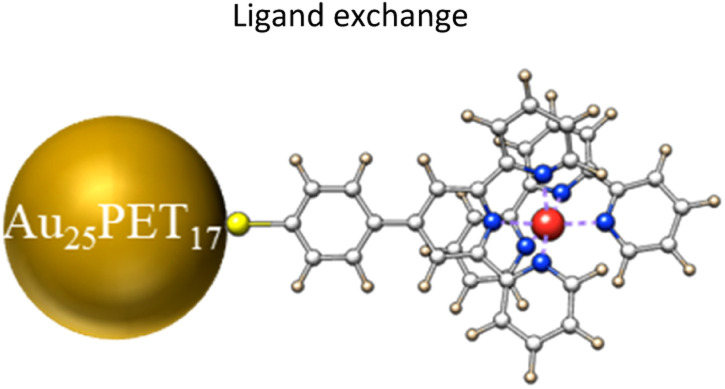	(*z* = 2+), M = Ru: 3965.9, M = Co: 3944.8, M = Fe: 3943.2, M = Ni: 3944.7, peak 2
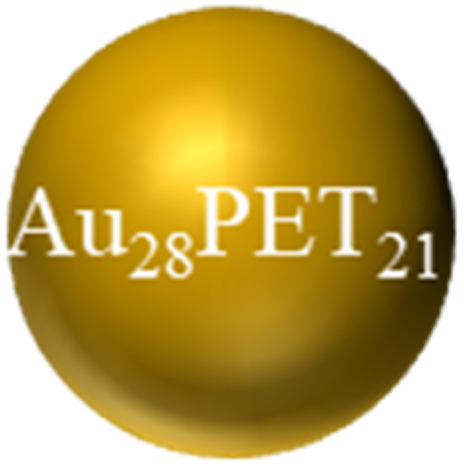	(*z* = 2+), 8396.8, peak 7
Ligand addition-Au_4_PET_4_	(*z* = 2+), M = Ru: 3366.1, M = Co: 3344.8, peak 1

**Fig. 4:**
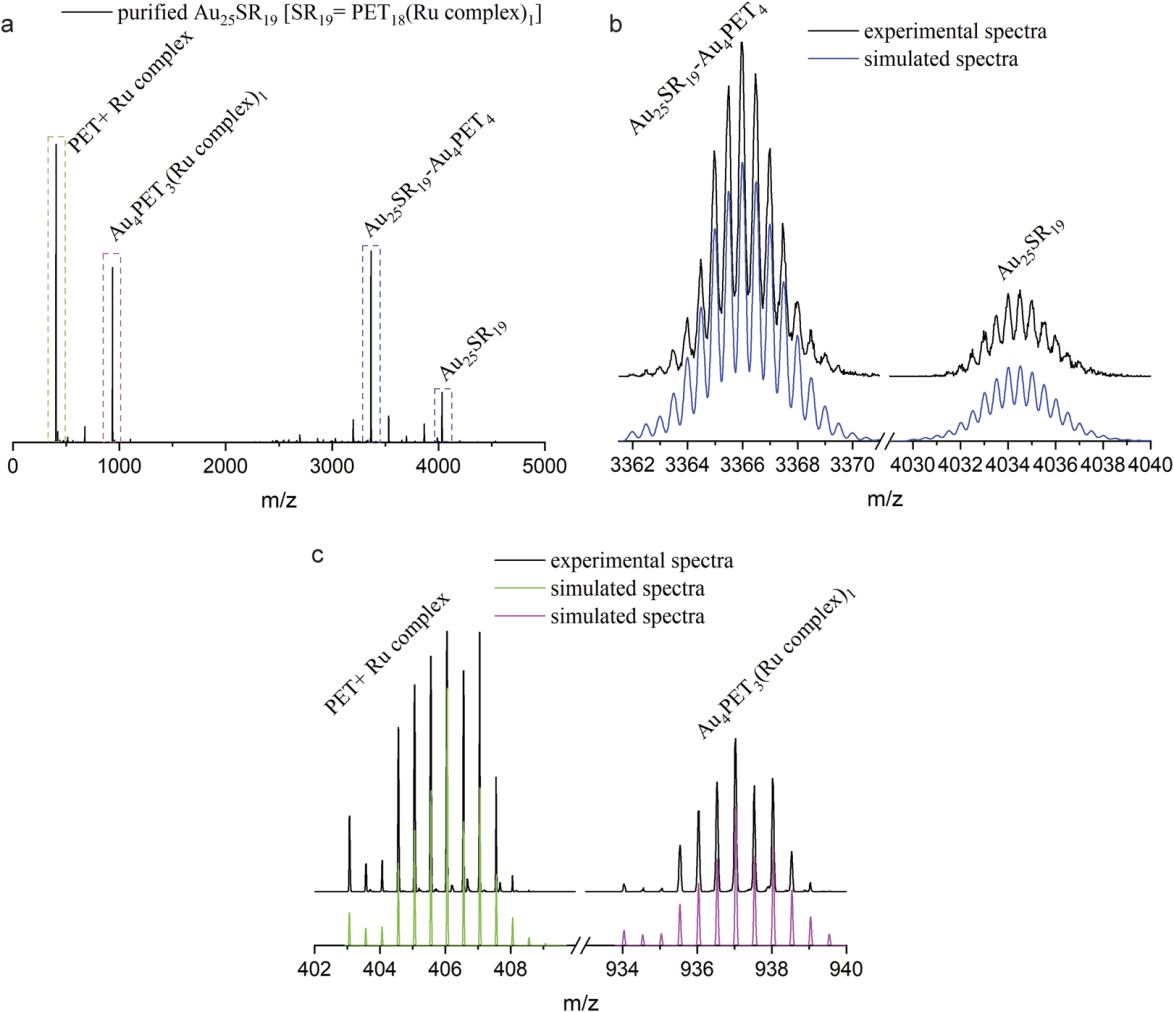
(a) ESI-MS of Au_25_SR_19_ [SR_19_ = PET_18_(Ru complex)_1_] after purification; (b) and (c): detailed isotopic peaks (in the square in a) and simulated isotopic patterns of labeled species, peak distance = 0.5.

A change of the ligand number while keeping the number of gold atoms constant will unavoidably affect the structure of the ligand shell and thus the properties of the whole NC. To study the photophysical properties, Au_25_SR_19_ [SR_19_ = PET_18_(Ru complex)_1_] was prepared at a NC/Ru complex ratio of 1 : 10 and purified by silica column chromatography with eluents dichloromethane and acetonitrile (3 : 1). HRESI-MS indicated good purity (Fig. 4a). The isotope patterns located at *m*/*z* = 4034.5 and *m*/*z* = 3366.1 (Fig. 4b) match perfectly with simulated spectra of Au_25_SR_19_ and its fragment after losing Au_4_PET_4_, which confirmed the molecular formula of Au_25_PET_18_(Ru complex)_1_. Fig. 4c shows some fragment peaks from Au_25_SR_19_ and the isotope patterns. The peak at *m*/*z* = 406.0 originates from the fragment PET + Ru complex ([C_8_H_9_S-SC_36_H_25_N_6_Ru]^2+^). In addition, the peak located at *m*/*z* = 937.1 is assigned to [Au_4_PET_3_(Ru complex)_1_]^2+^. Comparing the simulated isotopic patterns of Au_25_SR_19_ for covalent and noncovalent attachment of the metal complex with the experimental mass spectra (ESI, Fig. S10†) shows that the simulated isotopic patterns for the covalent attachment matches well the experiment, thus indicating a covalent binding of the metal complex to the cluster. The observed Au_4_SR_4_ fragment in ESI-MS may originate from staples (SR-Au-SR-Au-SR) of the NC *e.g.* from multistep rearrangement of two or more staples on the surface of the NC prior to fragmentation.^40,41^ A possible structure of Au_25_SR_19_ was already proposed by Xie *et al.*, which is characterized by the removal of one Au atom from the Au_13_ core and the formation of a Au_3_SR_4_ staple.^37^

**Fig. 5:**
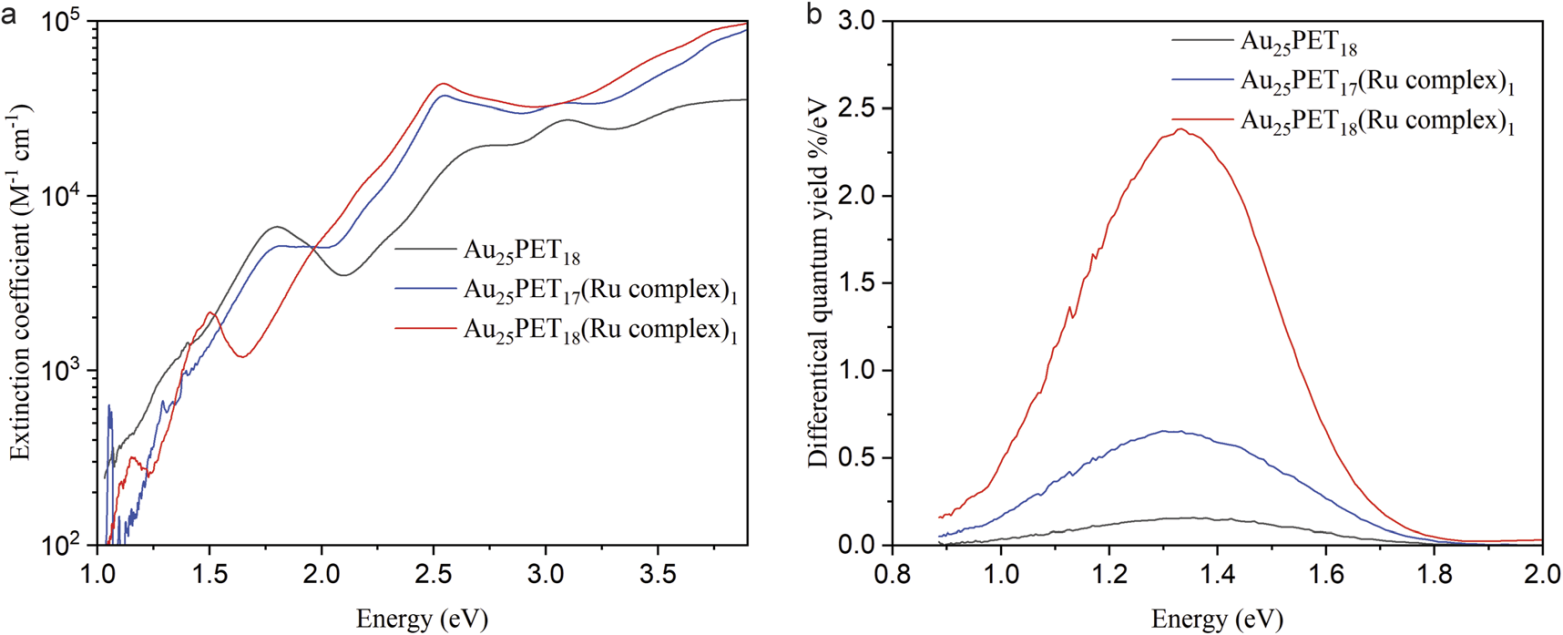
(a) The extinction coefficient for Au_25_PET_18_, Au_25_PET_17_(Ru complex)_1_, and Au_25_SR_19_ [SR_19_ = PET_18_(Ru complex)_1_]; (b) spectra of the differential luminescence quantum yield for Au_25_PET_18_, Au_25_PET_17_(Ru complex)_1_ and Au_25_PET_18_(Ru complex)_1._

The molar decadic extinction coefficient and differential luminescence quantum yield for Au_25_PET_18_, Au_25_PET_17_(Ru complex)_1_, and Au_25_SR_19_ [SR_19_ = PET_18_(Ru complex)_1_] are studied in Fig. 5a and b, respectively. As shown in Fig. 5a, the characteristic peaks from Au_25_SR_18_ still remained in the ligand-exchange product [Au_25_PET_17_(Ru complex)_1_], but disappeared after ligand addition [Au_25_PET_18_(Ru complex)_1_]. The intense peak at around 484.5 nm (2.56 eV) due to the Ru complex appeared in both Au_25_PET_17_(Ru complex)_1_ and Au_25_PET_18_(Ru complex)_1_, confirming that the Ru complex was associated with the NC.

The extinction coefficient for Au_25_PET_17_(Ru complex)_1_ and Au_25_PET_18_(Ru complex)_1_ after subtraction of the contribution from the Ru complex are shown in ESI, Fig. S11.† Au_25_PET_17_(Ru complex)_1_ exhibited very similar features compared with parent Au_25_PET_18_, while the spectrum of Au_25_PET_18_(Ru complex)_1_ completely changed, indicating that the electronic structure of Au_25_PET_18_(Ru complex)_1_ changed drastically compared to Au_25_PET_18_.

Interestingly, the luminescence spectra show very similar shape and peak position (Fig. 5b, ESI, Fig. S12† also shows emission spectra after normalization) for all three species Au_25_PET_18_, Au_25_PET_17_(Ru complex)_1_ and Au_25_PET_18_(Ru complex)_1_. The emission peak for pure Ru complex appears at 670 nm (1.85 eV, ESI, Fig. S12†). However, after binding to Au_25_ NCs, quenching of the chromophore fluorescence is observed, which can be due to energy transfer from the metal complex to the NC.^28^ The luminescence peak at 1.33 eV for Au_25_PET_18_, Au_25_PET_17_(Ru complex)_1_ and Au_25_PET_18_(Ru complex)_1_ probably stems from the core of the Au NCs, indicating that the core is not changing after ligand addition. However, Au_25_PET_18_(Ru complex)_1_ shows a drastic enhancement of quantum yield (Fig. 5b). Table 3 summarizes the luminescence quantum yields for these NCs. An increase of the luminescence quantum yield is observed for Au_25_PET_18_(Ru complex)_1_ compared to the initial Au_25_PET_18_. All samples exhibit a violation of Kasha's rule, as the luminescence shows up at higher energy than the lowest energy absorption band.

**Table 3:** Luminescence quantum yield of Au_25_PET_18_, Au PET_17_(Ru complex)_1_ and Au_25_PET_18_(Ru complex)_1_

	Quantum yield
Au_25_PET_18_	0.075%
Au PET_17_(Ru complex)_1_	0.32%
Au_25_PET_18_(Ru complex)_1_	1.05%

**Fig. 6:**
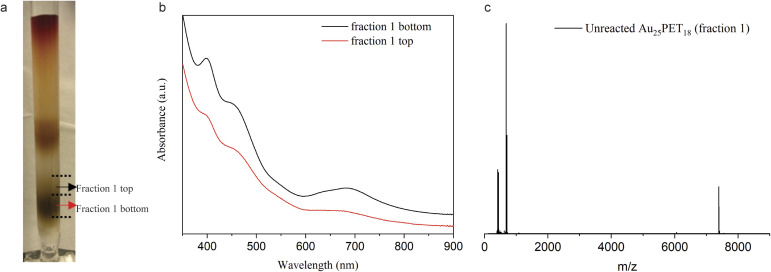
(a) Photograph of silica column after LER of Au_25_PET_18_ with Ru complex for 24 h at NC/complex ratio 1 : 4, fraction 2 contains both ligand exchange and ligand addition products; (b) UV-vis spectra of fraction 1 (top part and bottom part, as indicated in a); (c) ESI-MS of fraction 1 top part.

To further investigate the mechanism for the ligand addition reaction, unreacted Au_25_PET_18_ was separated from the reaction mixture, as shown in Fig. 6a, and studied. Fig. 6b shows UV-vis spectra of the top and bottom parts of fraction 1. According to these spectra, the bottom part can be assigned to neutral Au_25_PET_18_ (written as [Au_25_PET_18_]^0^), but in the UV-vis spectra of the top part, both peaks at around 400 nm and 700 nm were bleached, which indicates positively charged Au_25_PET_18_ (written as [Au_25_PET_18_]^+^).^42^ ESI-MS (Fig. 6c) was also applied to confirm the unreacted Au_25_PET_18_ of fraction 1 (top part). Since neutral Au_25_PET_18_ was used as the starting point for all ligand exchange reactions, however, both neutral and positively charged Au_25_PET_18_ were observed after ligand addition, we propose that [Au_25_PET_18_]^+^ should be the intermediate in the ligand addition reaction. Noteworthily, neutral Au_25_PET_18_ cannot be oxidized to [Au_25_PET_18_]^+^ on the silica column. Therefore, the transformation process from Au_25_SR_18_ to Au_25_SR_19_ is proposed as below: [Au_25_PET_18_]^0^ is oxidized to [Au_25_PET_18_]^+^, then [Au_25_PET_18_]^+^ NCs react with an external thiolate ligand to form a new isoelectric species, [Au_25_SR_19_]^0^, realizing the addition of one thiolate ligand to the original NC. The transformation mechanism is also consistent with the oxidization process proposed by Xie *et al.*^37^ In our work, the ligand addition reaction is dependent on the metal cations in the complexes, which indicates that metals play an important role in the process of ligand addition. We hypothesize that Ru and Co complexes are able to oxidize neutral Au_25_PET_18_ NCs to [Au_25_PET_18_]^+^ (in contrast to Fe and Ni complexes), so that the ligand addition reaction can take place easily. Moreover, the reported reduction potentials for the addition of the first electron to [M(tpy)_2_]^2+^ (M = Co, Ni, Fe, Ru; tpy = 2,2′:6′,2′′-terpyridine)^43,44^ indicate the following oxidant power of [M(tpy)_2_]^2+^: [Co(tpy)_2_]^2+^ > [Ru(tpy)_2_]^2+^ >[Fe(tpy)_2_]^2+^ >[Ni(tpy)_2_]^2+^. This observation is also consistent with our hypothesis that Co and Ru complexes have stronger tendency to oxidize the NC.

**Fig. 7:**
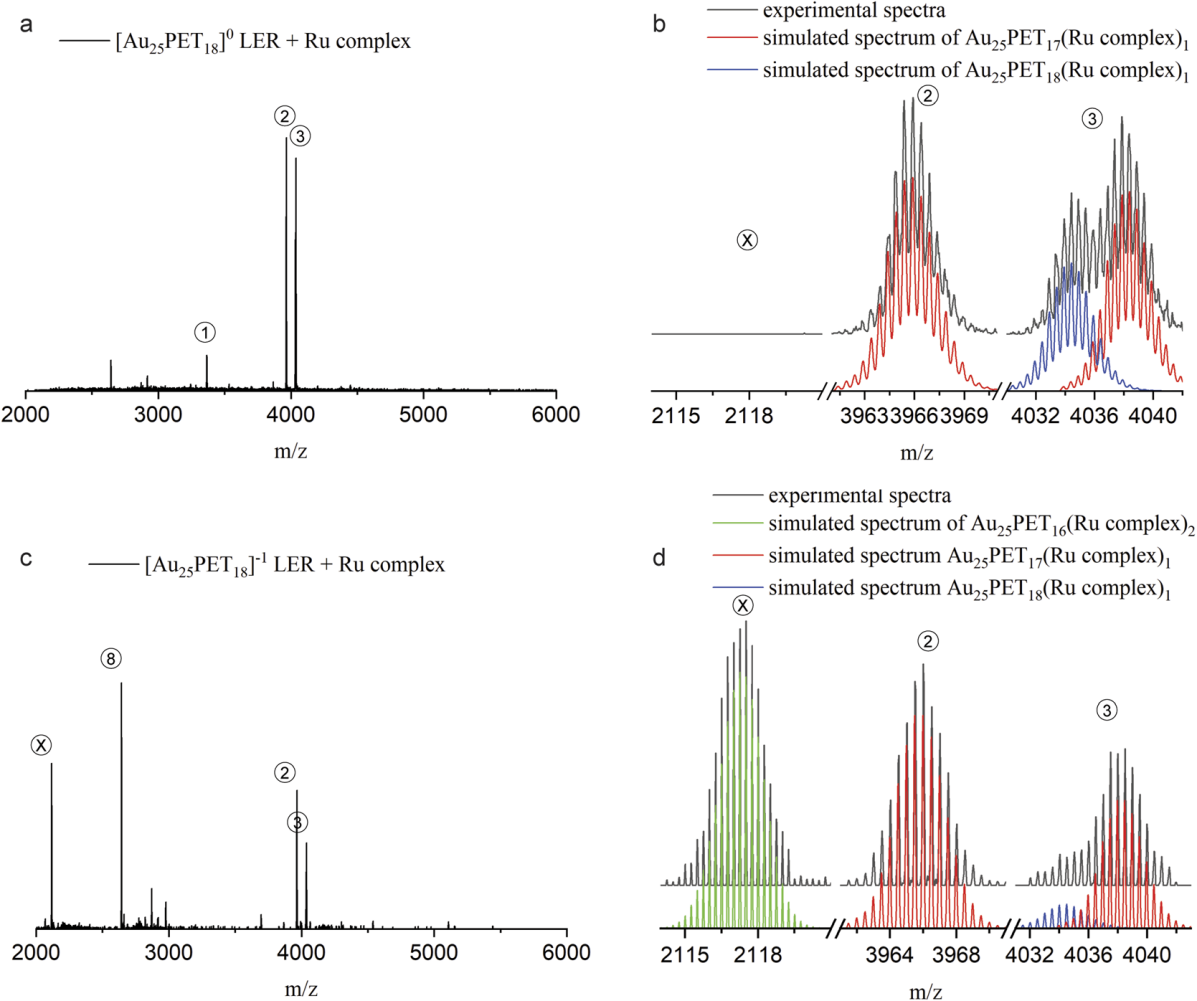
ESI-MS after LER started from neutral Au_25_PET_18_ (a and b) and negatively charged Au_25_PET_18_ (c and d) with Ru complex at NC/complex ratio 1 : 2 for 24 h.

Furthermore, we found that only one ligand at the surface could be replaced by metal complexes when neutral [Au_25_PET_18_]^0^ was used for LERs. In order to shed light on this interesting phenomenon, we performed LER starting from negatively charged Au_25_PET_18_ (written as [Au_25_PET_18_]^−1^) at a NC/complex ratio of 1 : 2. Fig. 7 shows HRESI-MS and detailed isotopic patterns after mixing either [Au_25_PET_18_]^0^ or [Au_25_PET_18_]^−1^ with the Ru complex and reacting for 24 h. As shown in Fig. 7a and b, products related with one ligand exchange (peak 2 and right part of peak 3) and addition of only one ligand (left part of peak 3) were observed when LER started form [Au_25_PET_18_]^0^. Interestingly, when [Au_25_PET_18_]^−1^ was used for LER with the Ru complex, the product of two ligand exchanges, Au_25_PET_16_(Ru complex)_2_, (peak X in Fig. 7c and d), was observed in addition to species resulting from one ligand exchange and ligand addition, respectively. The emerging peak X at *m*/*z* = 2117 (charge *z* = 4+) is assigned to [Au_25_PET_16_(Ru complex)_2_]^4+^ (molecular formula: (Au_25_C_200_H_194_N_12_S_18_Ru_2_)^4+^). The measured and simulated isotope patterns shown in Fig. 7b match very well. Another prominent peak at *m*/*z* = 2643.9 matches a NC with one ligand exchanged but with *z* = 3+, [Au_25_PET_17_(Ru complex)_1_]^3+^. Based on these interesting results, we hypothesize that the number of the metal complexes that can be exchanged to the NCs is strongly related to the charge of parent NCs. The Au_25_SR_18_ NC may be oxidized during LER with Ru complex, and easily accessible charge states for Au_25_SR_18_ are −1, 0, +1. Therefore, only one ligand can be substituted by metal complex on initially neutral Au_25_PET_18_, but two on initially negatively charged [Au_25_PET_18_]^−^. This could provide a promising method to precisely control the ligand-exchange number at the surface of NCs.

## Conclusions

In conclusion, Au_25_SR_19_ was successfully obtained by precisely adding one thiolated terpyridine-metal complex to Au_25_PET_18_ NCs during ligand exchange reaction at mild conditions. Moreover, we demonstrated that the reaction is metal dependent. Ru and Co complexes were favorable for ligand addition leading to Au_25_SR_19_, whereas Fe and Ni complexes promoted the exchange of one ligand. Also, NC/complex ratio and reaction time were studied, revealing that more metal complex and longer reaction time were advantageous for ligand addition on Au_25_. Therefore, the ligand addition process can be easily and precisely controlled. HRESI-MS also gave some hints concerning the structure of Au_25_SR_19_. The new NC showed different electronic structure and strongly enhanced luminescence compared with pristine Au_25_PET_18_. The manipulation of the number of ligands at a given Au NCs provides additional possibilities for the diversification of NCs and to change their physiochemical properties, thus boosting the application of Au NCs. More interestingly, only one ligand can be replaced on neutral Au_25_PET_18_ but two on negatively charged Au_25_PET_18_ during LER with metal complexes, which provides us with a novel methodology to precisely control the ligand-exchange number at the surface of NCs.

Experimental section shown in ESI.†

## Data availability

The original data leading to this publication are available at https://doi.org/10.5281/zenodo.8079142.

## Author contributions

T. B. and A. Z. conceived the idea of the research. J. Z. performed all of the experiments, analysed data and wrote the manuscript with the help of Y. W., A. Z. and T. B. The photophysical experiment was conducted with the help of A. R. All authors contributed to editing the manuscript.

## Conflicts of interest

There are no conflicts to declare.

## Supplementary Material

SC-014-D3SC01177A-s001
