# Supplementary material for: Engineering ligand chemistry on Au25 nanoclusters: from unique ligand addition to precisely controllable ligand exchange
Source: Chem Sci. 2023 Jun 22;14(28):7665–74. doi: 10.1039/d3sc01177a (PMC10355100; doi:10.1039/d3sc01177a)
Supplement: SC-014-D3SC01177A-s001 [file SC-014-D3SC01177A-s001.pdf]

Electronic Supplementary Material (ESI) for Chemical Science.
This journal is © The Royal Society of Chemistry 2023

Supplementary information

Engineering ligand chemistry on Au25 nanocluster: From unique ligand addition to precisely controllable ligand exchange

*Jiangtao Zhao [a], Abolfazl Ziarati *[a], Arnulf Rosspeintner [a], Yanan Wang [b] and Thomas Bürgi *[a]*

[a]Department of Physical Chemistry, University of Geneva, 30 Quai Ernest-Ansermet, 1211 Geneva 4, Switzerland.
[b]Department of Chemical Engineering, University of Michigan, Ann Arbor, MI, USA.

Email: Thomas.Buergi@unige.ch; Abolfazl.Ziarati@unige.ch

Materials

All chemicals were purchased from commercial suppliers and used without any further treatment.

Synthesis of $Au_{25}PET_{18}$ nanoclusters

$[Au_{25}PET_{18}]^{-1}$ was initially prepared and oxidized subsequently by silica gel column to form $[Au_{25}PET_{18}]^{0}$. Typically, 1g $HAuCl_4 \cdot 3H_2O$ and 1.666g TOAB were dissolved in 250mL THF and vigorously stirred for 15min in a 1L-round-bottom flask. The color of the solution changed from yellow to red. Afterwards, 1.7mL 2-phenylethanethiol was added slowly and the solution was stirred for 30min, the color changed from red to yellow and colorless. Subsequently, 0.96g NaBH4 dissolved in 50mL ice-cold MilliQ water was added to the solution rapidly, and the color changed to black immediately. Then the reaction was continued for 48h. The obtained solution was concentrated by rotary evaporator, resulting in a brown oil phase covered by water. In order to remove the free ligand, an excess amount of methanol was added to the flask, filtrated, dissolved the substance in DCM and dried in vacuum again. This process was repeated at least three times and $[Au_{25}PET_{18}]^{-1}$ was obtained. $[Au_{25}PET_{18}]^{0}$ was produced by further oxidation in silica gel column eluted by DCM.

Fabrication of and metal complex (Metal= Ru, Fe, Co, Ni)

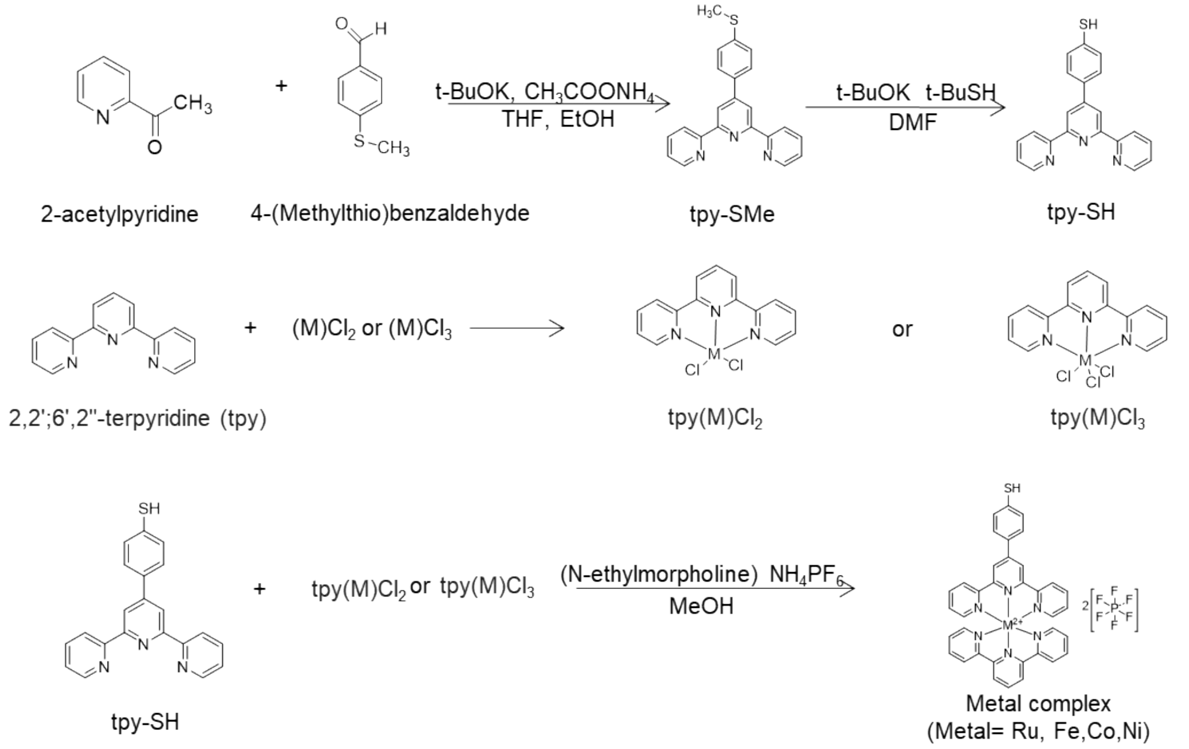

Scheme 1. Synthesis procedure of metal complex

Synthesis of 4'-[4-(Methylthio)phenyl]-2,2';6',2''-terpyridine (tpy-SMe)[1]

In a glovebox, a flame-dried two-necked flask (500mL) was charged with 5.6g potassium tert-butoxide (t-BuOK) and 75mL anhydrous THF. After vigorously stirring for half hour, 3.55mL 2-acetylpyridine was added dropwise by syringe, and the color turned yellow. Then 2mL 4-(methylthio)benzaldehyde was injected to the flask slowly and the color changed to ruby red during stirring. The mixture was continued reacting for about 60h. Afterwards, 38g ammonium acetate suspended in 270mL ethanol was added, followed by refluxing at inert atmosphere for 5h and the color changed to yellow. Subsequently, the reaction mixture was cooling down and the solvent was concentrated to approximately 150mL, followed by filtering to get a yellow solid. The solid was washed by warm ethanol, filtered again and recrystallized in DCM/Ethanol mixture. The filtration from the reaction was repeated according to the above procedure to obtain more product. ^{1}H NMR and ^{13}C NMR Spectra of tpy-SMe were shown in Supplementary Figure S6. ^{1}H NMR (400 MHz, $CDCl_3$): $\delta = 2.55$ [3H; SCH_3], 7.36 [2H; $tpySCH_3(5,5^{II})$], 7.38 [2H; Ph(3,5)], 7.86 [2H; Ph(2,6)], 7.88 [2H; $tpySCH_3(4,4^{II})$], 8.67 [2H; $tpySCH_3(3,3^{II})$, 8.73 [2H; $tpySCH_3(3^{I},5^{I})$, 8.74 [2H; $tpySCH_3(6,6^{II})$]. ^{13}C NMR (100.6 MHz, $CDCl_3$): $\delta = 15.5$ [CH_3], 118.4 [CH; $tpySCH_3(3^{I},5^{I})$], 121.4 [CH; $tpySCH_3(3,3^{II})$], 123.8 [CH; $tpySCH_3$ $(5,5^{II})$], 126.5 [CH; Ph(3,5)], 127.6

[CH; Ph(2,6), tpy($5,5^{II}$)], 134.9 (C), 136.8 [CH; tpySCH_3 ($4,4^{II}$)], 140.1 (C), 149.1 [CH; tpySCH_3 ($6,6^{II}$)], 149.5 (C), 156.0 (C), 156.2 (C).

Preparation of 4'-[4-(mercapto)phenyl]-2,2';6',2''-terpyridine (tpy-SH)

500mg tpy-SMe, 1.2g t-BuOK and 10mL anhydrous DMF were added to a completely dried two-necked flask (25mL). After stirring for several minutes, 0.8mL t-BuSH was added to the flask. Then freeze-pump-thaw was repeated three times to remove the gas and the suspension was refluxed for 12h at 130 °C and 3h at 150°C. After cooling in an ice-bath, the red solution was mixed with 30mL MilliQ water followed by filtration to get the filter liquor. Subsequently, 75mL saturated ammonium chloride aqueous solution was added and yellow precipitation appeared. The precipitation was collected by filtering and washed several times with water and ethanol. ^{1}H NMR and ^{13}C NMR spectra of tpy-SH is shown in Supplementary Figure S7. ^{1}H NMR (400 MHz, $CDCl_3$): δ = 3.58 [s, 1H; SH], 7.36 [2H; tpySH($5,5^{II}$)], 7.41 [2H; Ph(3,5)], 7.81 [2H; Ph(2,6)], 7.89 [2H; tpySH ($4,4^{II}$)], 8.68 [2H; tpySH($3,3^{II}$)], 8.71 [2H; tpySH($3^{I},5^{I}$)], 8.74 [2H; tpySH($6,6^{II}$)]; ^{13}C NMR (100.6 MHz, $CDCl_3$, ppm): δ = 118.6 [CH; tpySH($3^{I},5^{I}$)], 121.6 [CH; tpySH($3,3^{II}$)], 124.0 [CH; tpySH($5,5^{II}$)], 128.0 [CH; Ph(2,6)], 129.5 [CH; Ph(3,5)], 132.7 (C), 135.5 (C), 137.2 [CH; tpySCH3($4,4^{II}$)], 148.9 [CH; tpySCH_3($6,6^{II}$)], 149.5 (C), 155.7 (C), 155.9 (C). MS: ((DIP) EI-MS, positive polarity), m/z = 308.16 [M-SH], 341.01 [M].

Synthesis of tpy(M)Cl_2 or tpy(M)Cl_3

tpy(Ru)Cl_3 and tpy(Ni)Cl_2 were prepared by the same method. tpy (233mg) in 10 mL of ethanol was added dropwise to $RuCl_3 \cdot 3H_2O$ (261mg) or $NiCl_2 \cdot 6H_2O$(240mg) solution (10mL ethanol), followed by refluxing for 4h. The final product was collected by filtering and washed by ethanol and diethyl ether.

tpy(Fe)Cl_2 and tpy(Co)Cl_2 were fabricated in inert atmosphere. Typically, 233mg tpy and 126mg $FeCl_2$ in 20mL anhydrous THF were stirred overnight, then 50mL pentane was added to precipitate purple product of tpy(Fe)Cl_2. To obtain tpy(Co)Cl_2, 233mg tpy and 130mg $CoCl_2$ were dissolved in 20mL anhydrous THF and stirred overnight, the resulting mixture was concentrated in vacuo, filtrered and washed by ethanol and diethyl ether.

Synthesis of metal complex[2]

20 mg tpy-SH was added to the suspension of tpy(M)Cl_2 or tpy(M)Cl_3 (1.0 equiv) in methanol (12mL). Three drops of N-ethylmorpholine were added (for Ru complex) to the mixture, followed by flowing

Ar gas for 1h to keep inert atmosphere. Then refluxing was applied overnight. After cooling down, the mixture was filtered through filter paper. After that excess amount of ammonium hexafluorophosphate was added to precipitate all Ru complexes. The solid was collect by filtration and washed with water. Metal complex was purified by silica gel column chromatography (eluents: acetonitrile/0.2M aqueous potassium nitrate solution 4:1). The second fraction was collected and the solvent was evaporated. Then excess aqueous NH_4PF_6 solution was added to exchange the counterions. Finally, the product was obtained by filtration and washed with excess water, ethanol and diethyl ether. ^1H NMR spectra of Ru complex is shown in Supplementary Figure S2. ^1H NMR (400 MHz, CD_3CN): δ = 7.19 [4H; tpySH($5,5^{II}$), tpy($5,5^{II}$)], 7.37 [2H; tpy($6,6^{II}$)], 7.42 [2H; tpySH($6,6^{II}$)], 7.94 [4H; tpySH($4,4^{II}$), tpy($4,4^{II}$)], 8.03 [2H; Ph(3,5)], 8.26 [2H; Ph(2,6)], 8.44 [1H; tpy(4^I)], 8.51 [2H; tpy($3,3^{II}$)], 8.65 [2H; tpySH($3,3^{II}$)], 8.77 [2H; tpy($3^I,5^I$)], 9.02 [2H; tpySH($3^I,5^I$)].

Characterizations

UV-Vis spectra were measured on JASCO V-670 UV-VIS-NIR spectrophotometer. Bruker 400 MHz was used to collect ^1H-NMR and ^{13}C NMR spectra. Low-resolution ESI-MS was recorded by an ESI-MS instrument, API 150EX from AB/MDS Sciex. High-resolution (HR) ESI-MS was studied by QSTAR Pulsar (QqTOF) instrument, with positive polarity. The experimental parameters of HR ESI-MS were the following: ESI voltage = 5500 V (positive), curtain gas (N_2) = 25 psi, declustering and focusing lenses: DP = 60 V, DP2 = 10 V, FP =80 V, Bin = 1 (automatic signal smoothing).

Luminescence spectra and quantum yields were measured with a FluoroLog 3-1iHR (PMT R5509-73) in combination with an integrating sphere (G8, GMP). The luminescence spectra were corrected for the instrument sensitivity according to the fluorimeter manufacturer. The correction function of the integrating sphere was obtained comparing the fluorimeter lamp profile using the integrating sphere and a Spectralon reflector as reference. Contributions of the inherent emission from the integrating sphere were subtracted (scaled for the difference in scattered light between empty and loaded sample).

Ligand exchange reactions

Purified neutral $Au_{25}PET_{18}$ in DCM was mixed with metal complex (in acetonitrile) at different Au NCs/complex molar ratios (final nanocluster concentration 1mg/mL) and stirred in glovebox. Ligand

exchange reactions took place at room temperature and HRESI-MS of the samples after reaction for different time was performed without purification (unless otherwise noted). Time-course UV-Vis absorption spectra after LER with different metal complexes for different time at nanocluster/complex ratio 1:2 was shown in Supplementary Figure S8.

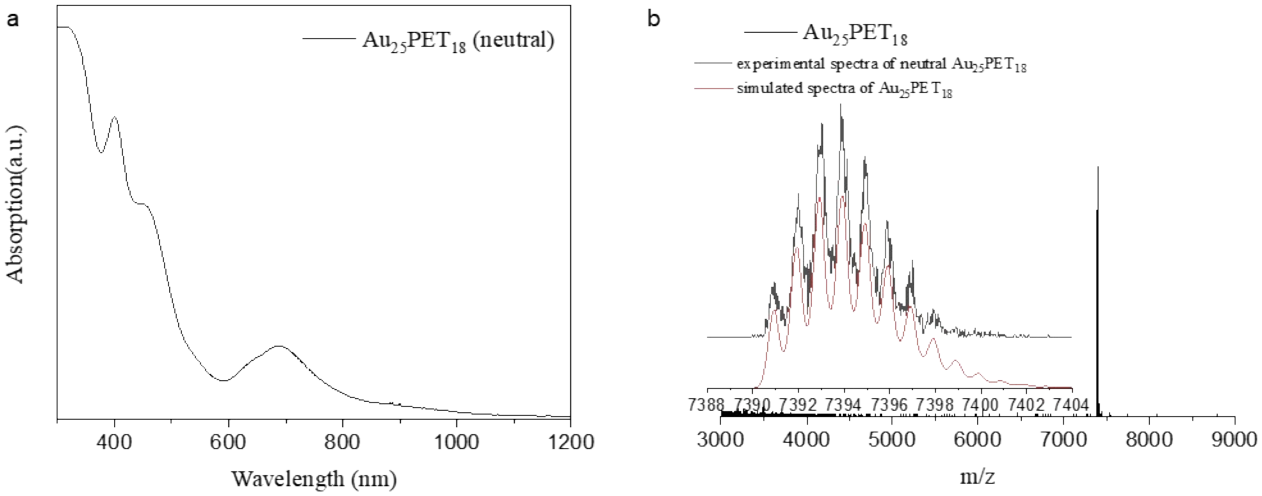

Supplementary Figure S1. (a) UV-Vis absorption spectrum of $Au_{25}PET_{18}$ in DCM and (b) HR-ESI MS of $Au_{25}PET_{18}$ and comparison of experimental and simulated isotopic patterns of $Au_{25}PET_{18}$.

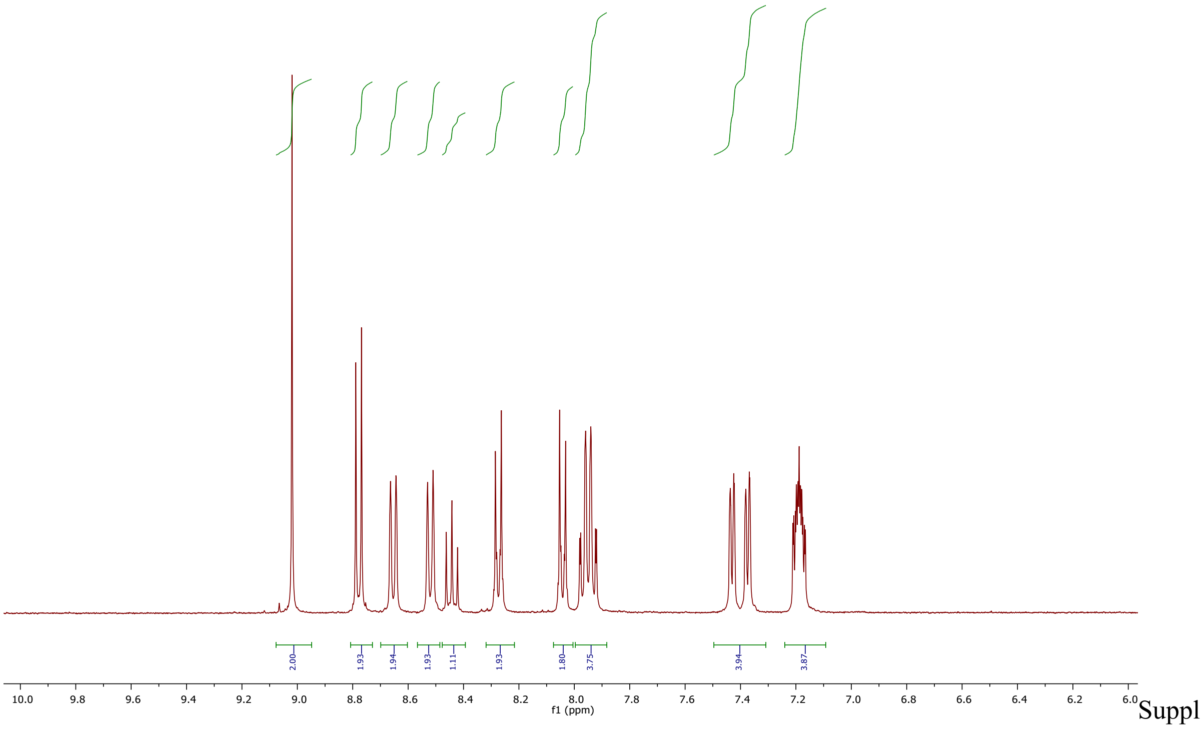

Supplementary Figure S2. ^{1}H-NMR of as-prepared Ru complex in CD_3CN.

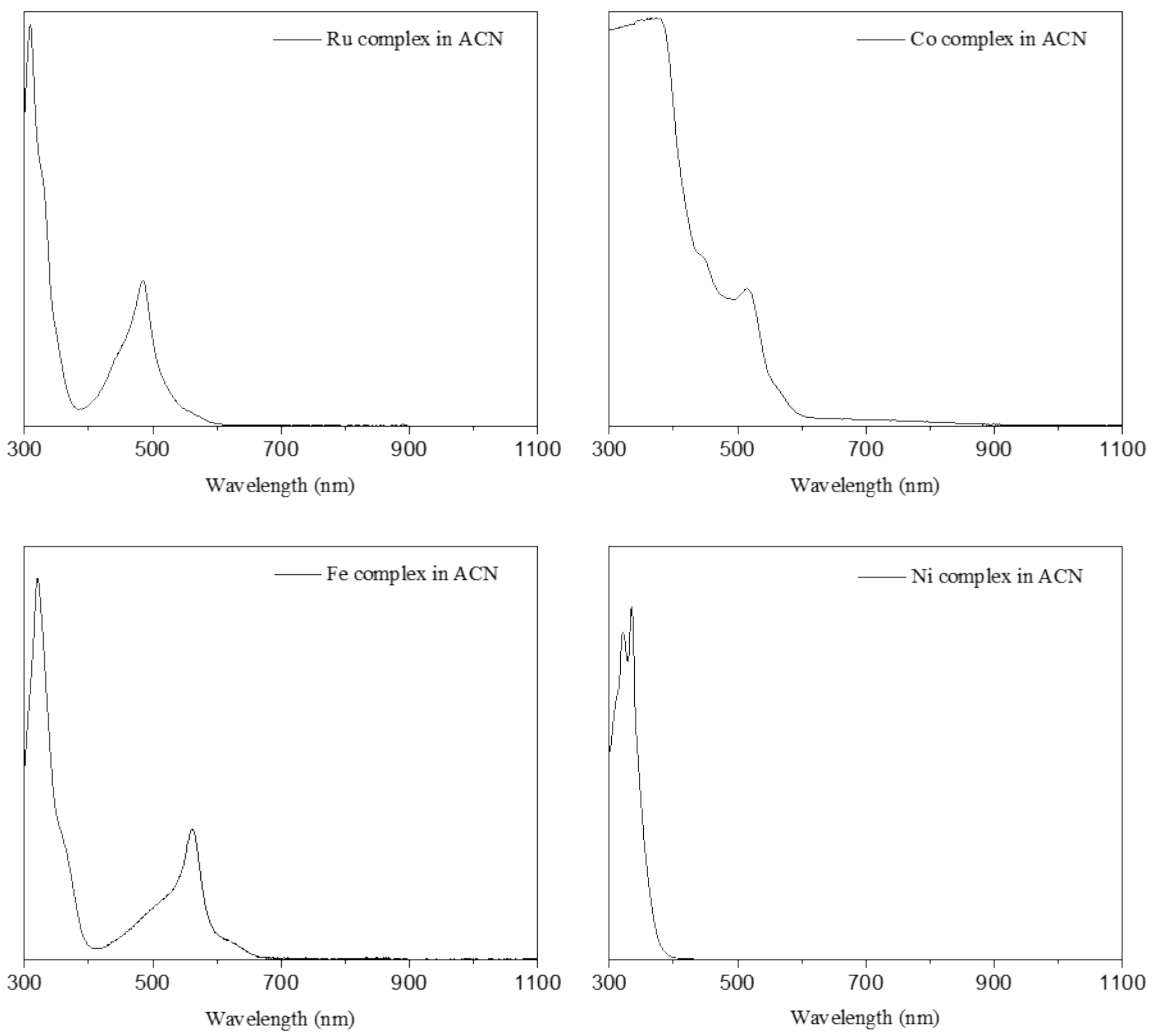

Supplementary Figure S3. UV-Vis absorption spectra of as-prepared metal complexes in Acetonitrile (ACN).

Supplementary Table S1. ESI-MS of as prepared metal complex in Acetonitrile.

	m/z $[(M\text{-}2PF_6^-)^{2+}]$	m/z $[(M\text{-}PF_6^-)^{+}]$
Ru complex	337.6 (Cal. 337.88)	820.0(Cal. 820.73)
Co complex	316.3 (Cal. 316.82)	777.3 (Cal. 778.6)
Fe complex	314.8 (Cal. 315.27)	774.3 (Cal. 775.51)
Ni complex	316.1 (Cal. 316.70)	777.4 (Cal. 778.36)

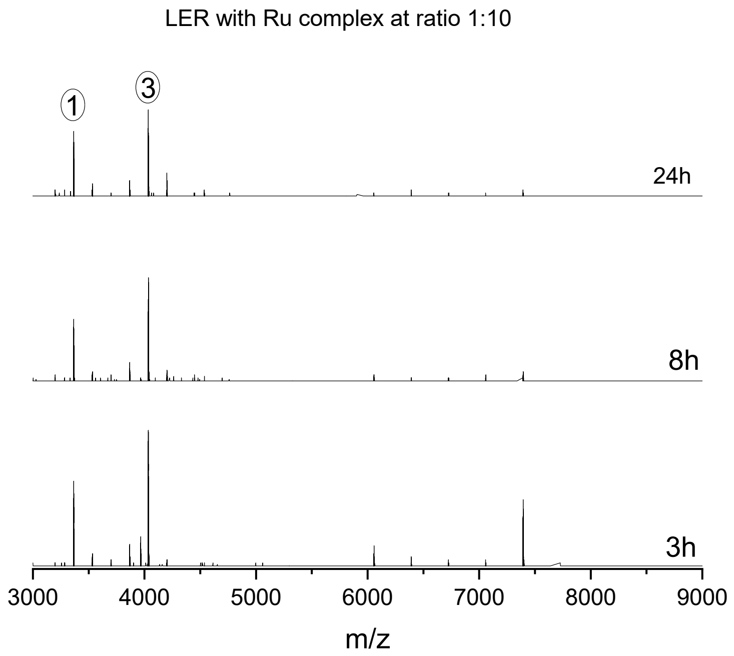

Supplementary Figure S4. ESI-MS after ligand exchange between $Au_{25}PET_{18}$ and Ru complex at cluster/Ru complex ratio 1:10.

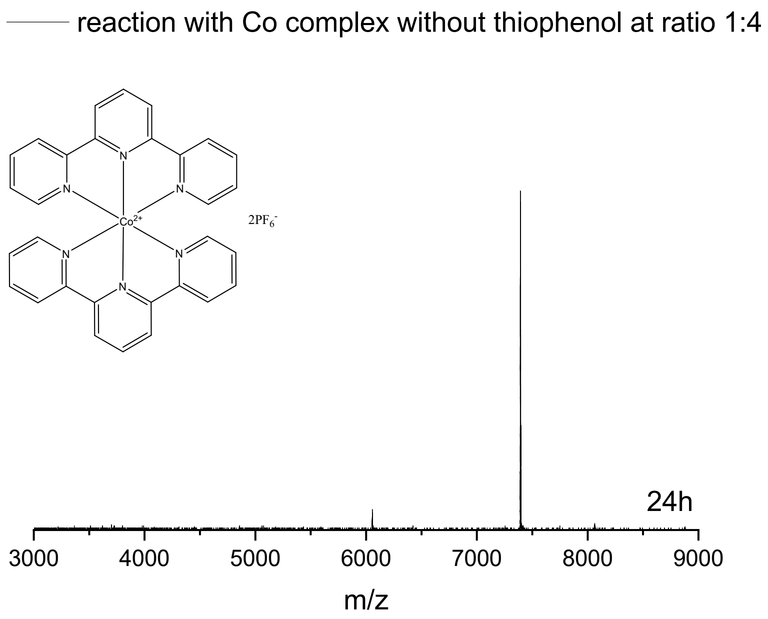

Supplementary Figure S5. ESI-MS after reaction between $Au_{25}PET_{18}$ and above complex for 24h.

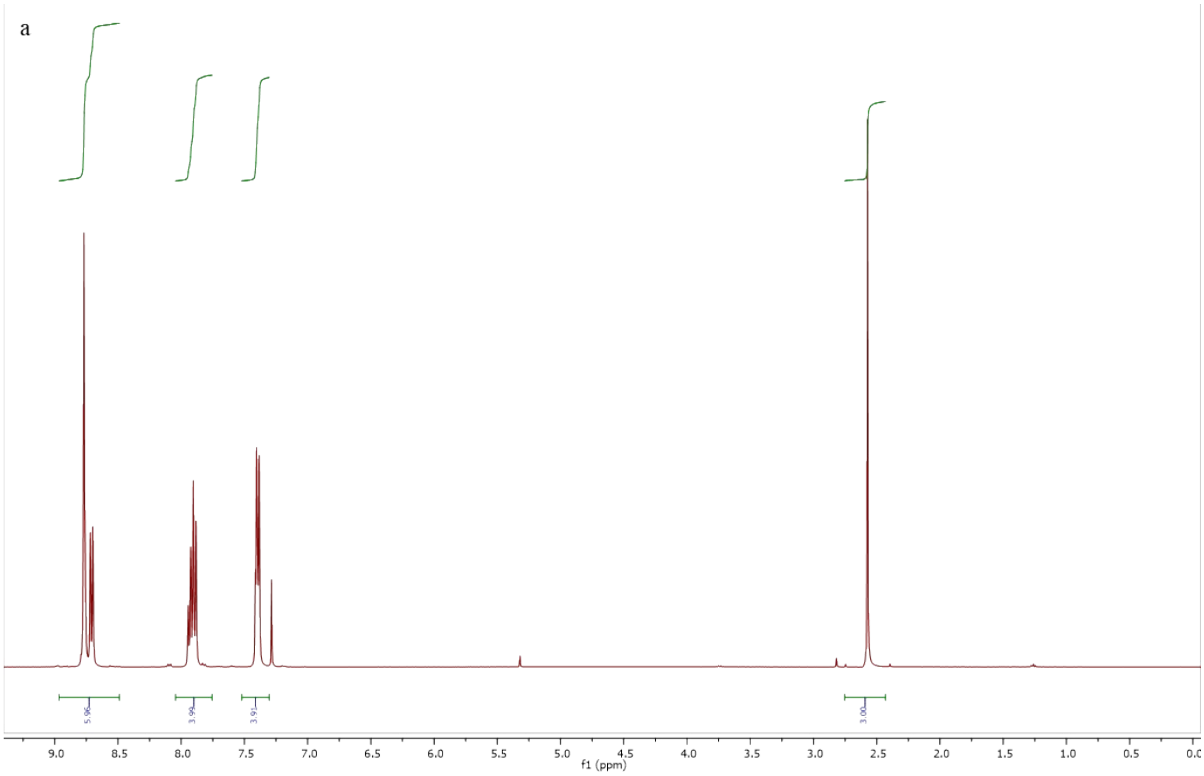

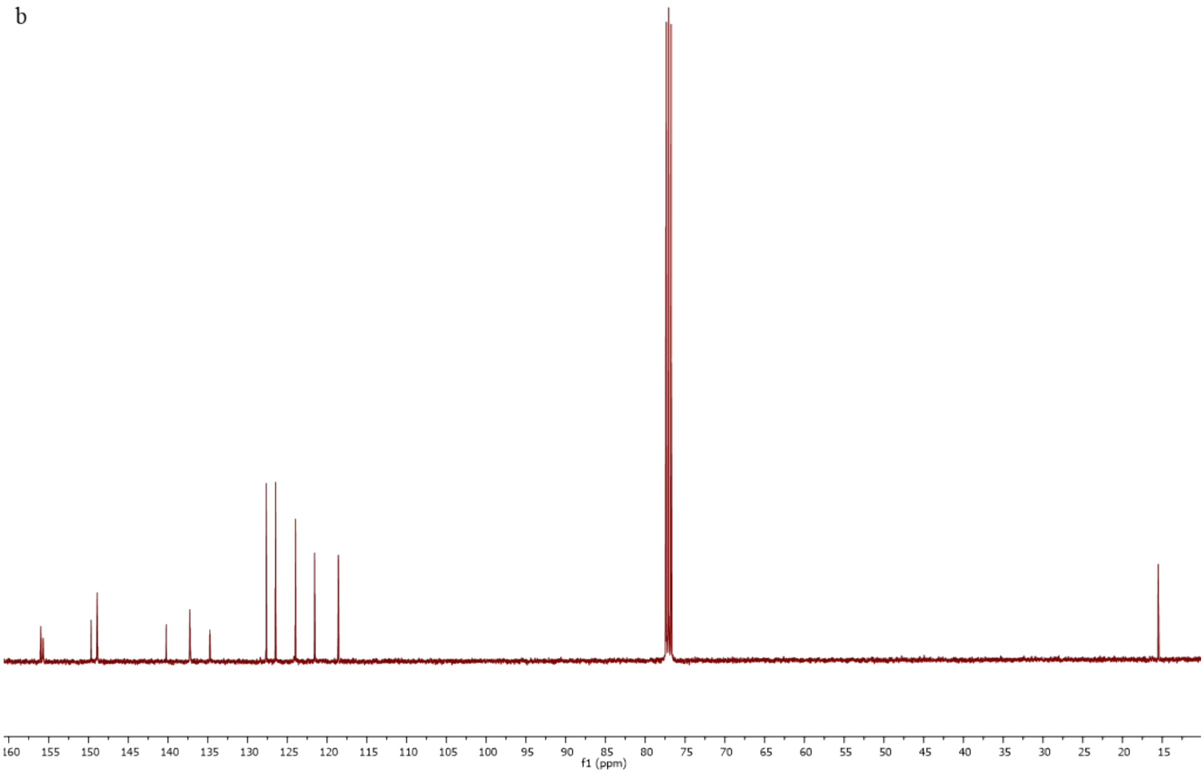

Supplementary Figure S6. ^{1}H-NMR (a) and ^{13}C NMR (b) of tpy-SMe in $CDCl_3$.

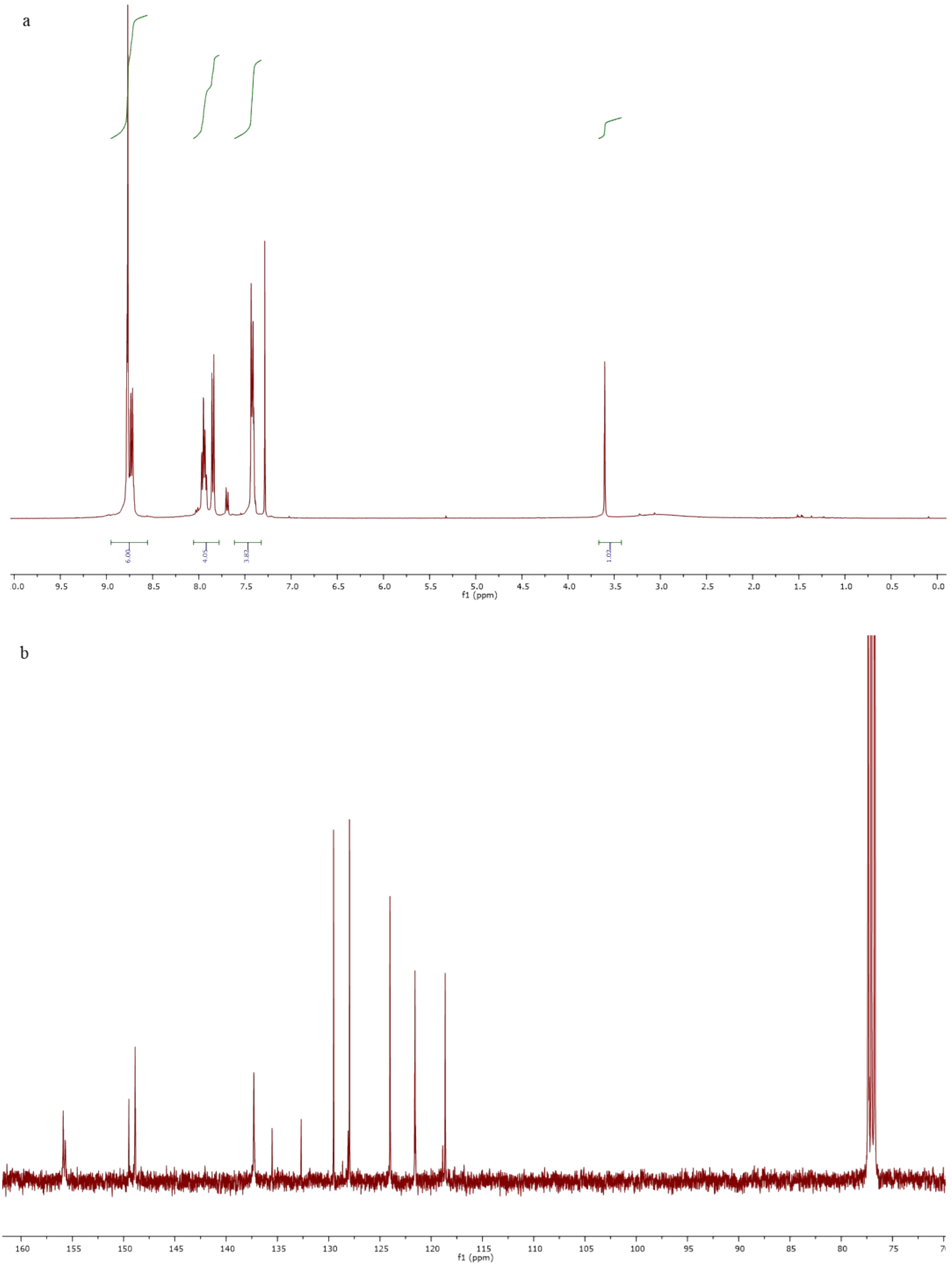

Supplementary Figure S7. ^{1}H-NMR (a) and ^{13}C NMR (b) of tpy-SH in $CDCl_3$.

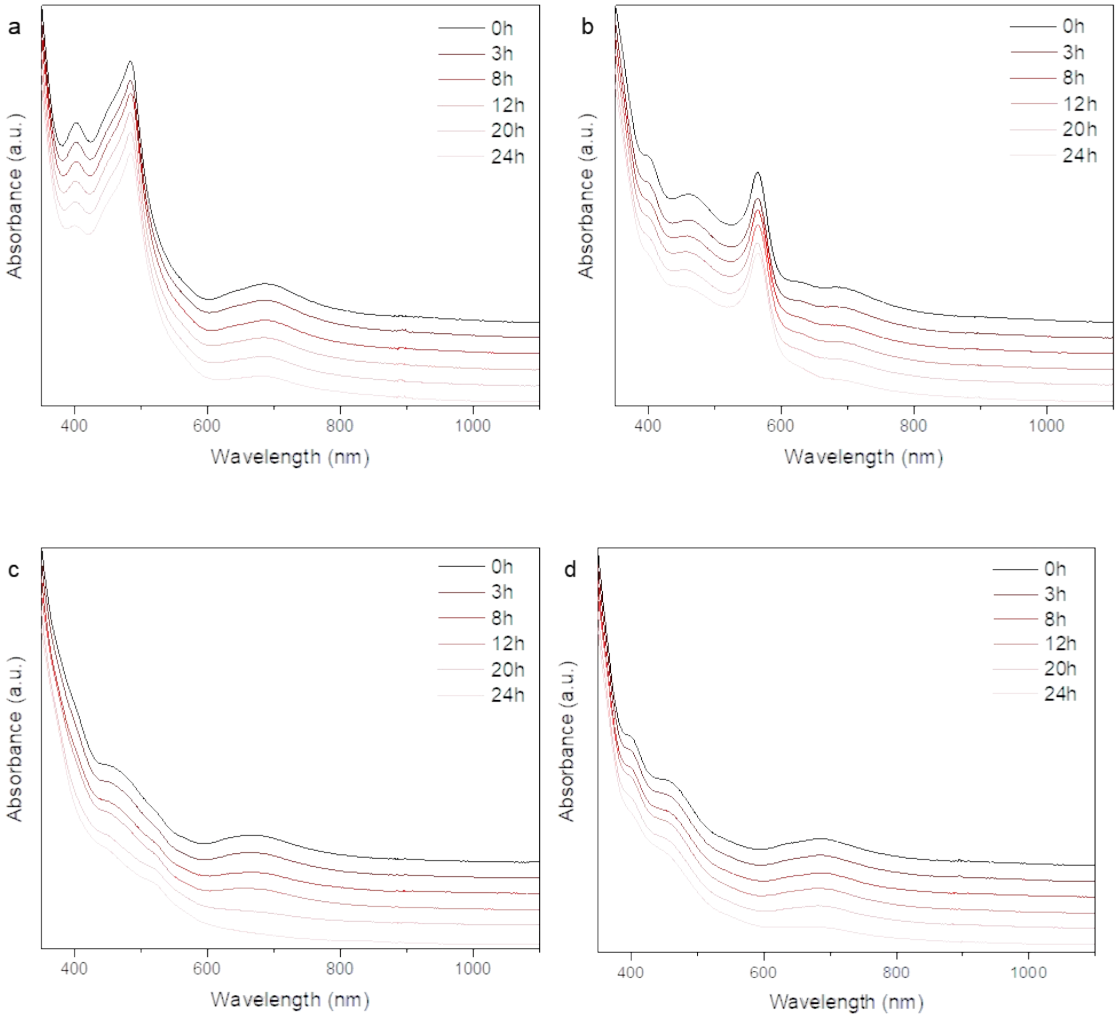

Supplementary Figure S8. Time-course UV-Vis absorption spectra after LER with Ru complex(a), Fe complex (b), Co complex (c) and Ni complex (d) at cluster/complex ratio 1:2. Spectra were offset for clarity. Characteristic peaks from $Au_{25}PET_{18}$ become weaker during the reaction.

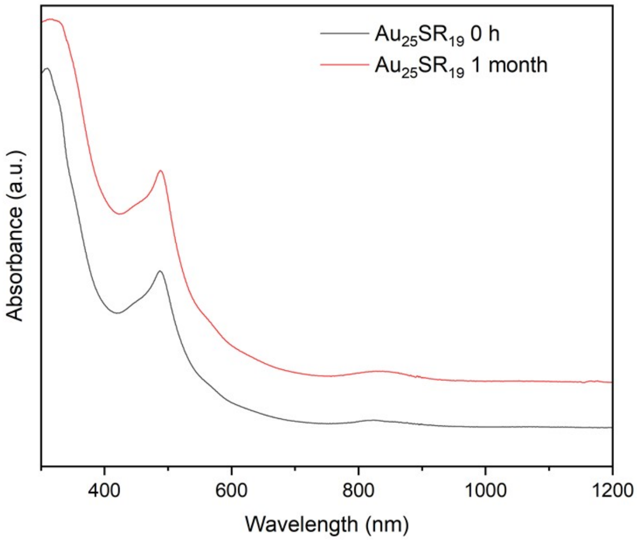

Supplementary Figure S9. (a) UV-vis absorption spectra of $Au_{25}SR_{19}$ solution after 30 days in fridge. No obvious changes in absorption spectra indicates the high stability.

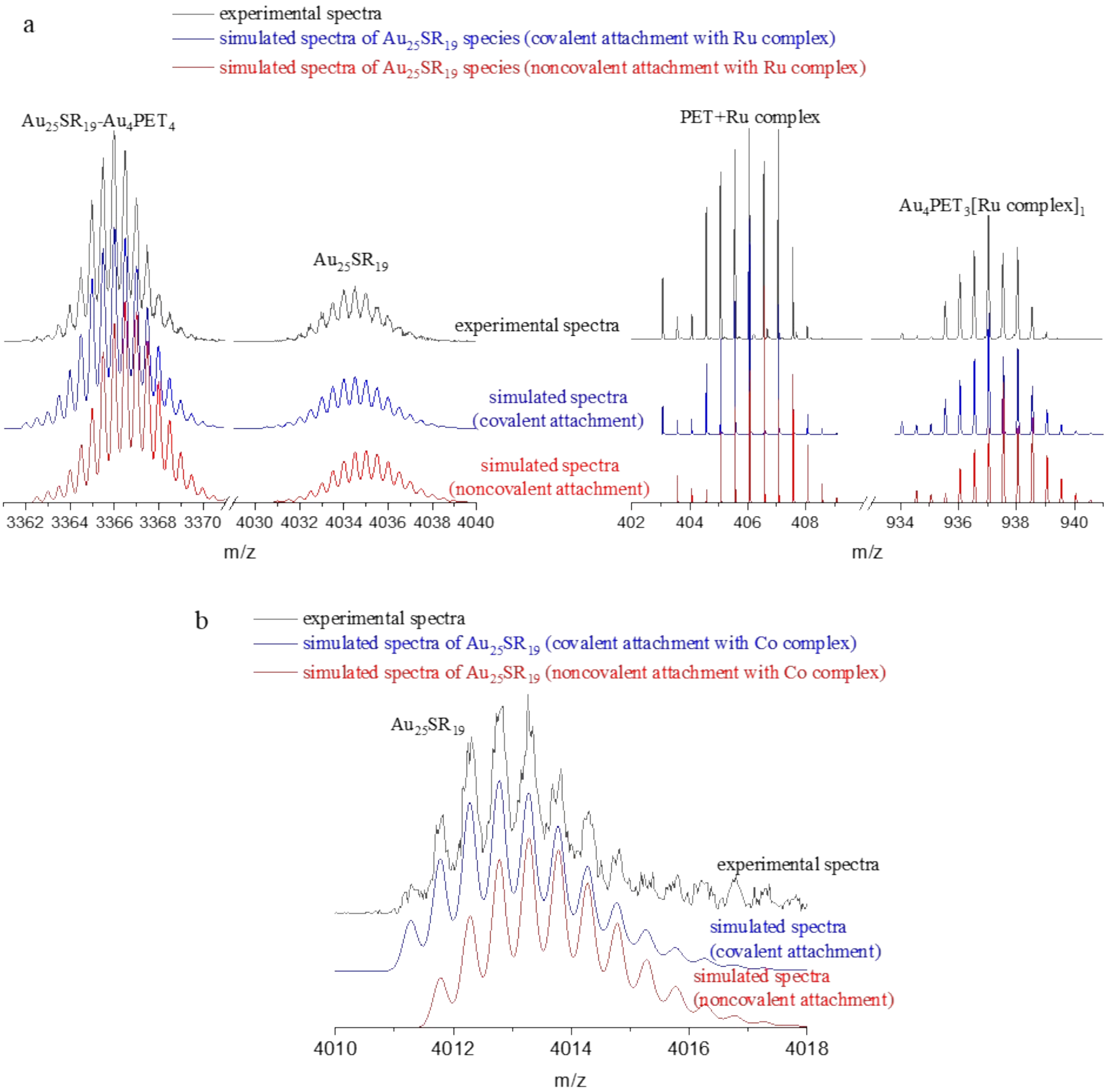

Fig. S10. (a) Comparison of experimental and simulated spectra of $Au_{25}SR_{19}$ and its fragments for covalent and noncovalent attachment of the Ru complex. (b) Comparison of experimental and simulated spectra of $Au_{25}SR_{19}$ for covalent and noncovalent attachment with Co complex.

The comparison between experimental isotopic patterns and simulated ones of $Au_{25}SR_{19}$ and its fragments for covalent and noncovalent attachment of the Ru complex is shown in Fig. S10a. It demonstrates that the simulated spectra of the species with noncovalent attachment of the Ru complex is positively shifted by 0.5 units compared to that of covalently bonded Ru complex. The comparison indicates that Ru complex is covalently bonded to the NC. Fig. S10b shows the comparison of experimental isotopic patterns and simulated spectra of $Au_{25}SR_{19}$ for covalent and noncovalent attachment of Co complex; it also reveals that Co complex is covalently attached to the cluster. These results are in accordance with the conclusion drawn from Fig. S5.

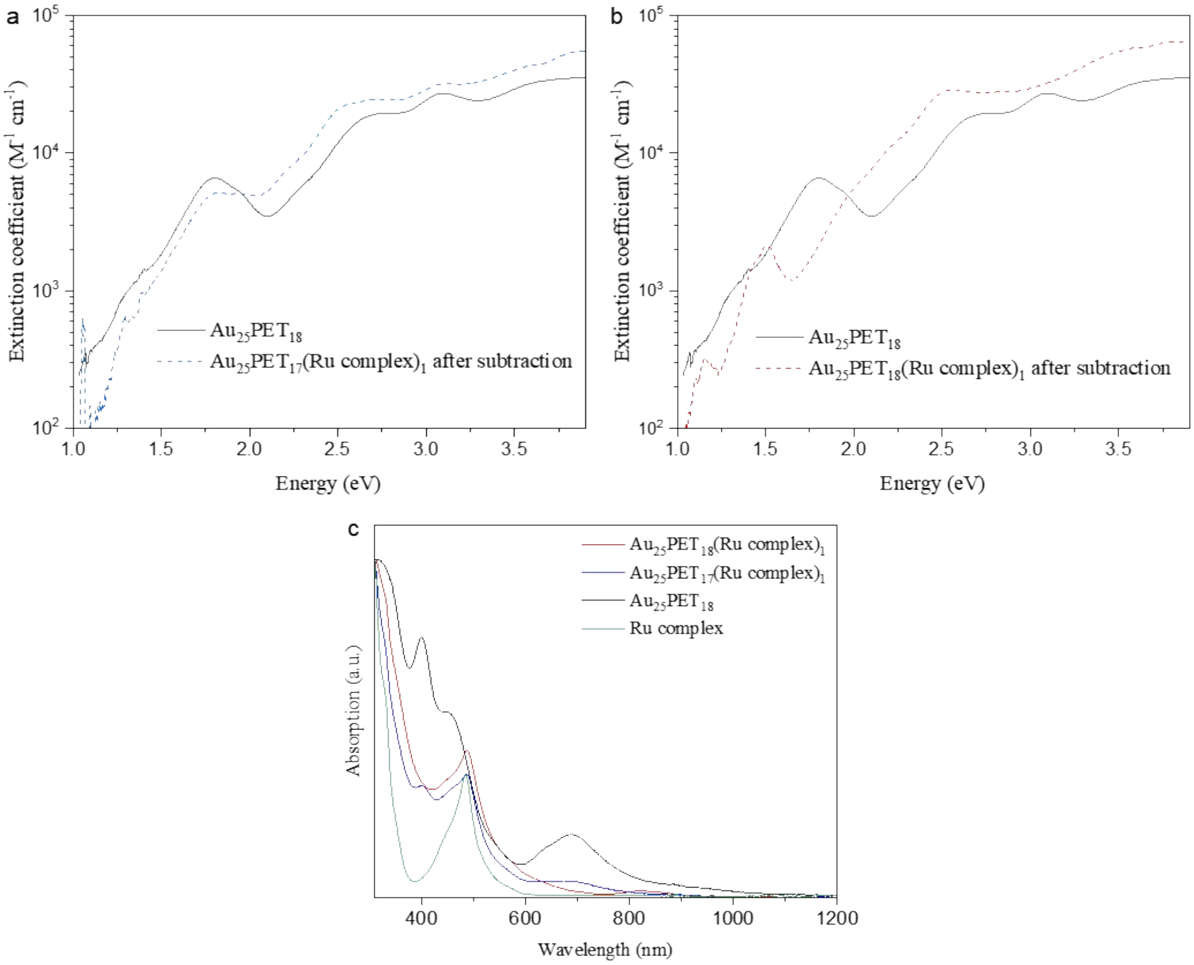

Supplementary Figure S11. The comparison of spectral extinction coefficient after subtraction of the Ru complex for $Au_{25}PET_{17}$(Ru complex)$_1$ and $Au_{25}PET_{18}$ (a), $Au_{25}SR_{19}$ [SR_{19}=PET_{18}(Ru complex)$_1$] and $Au_{25}PET_{18}$ (b), and UV-Vis absorption spectra of $Au_{25}PET_{18}$(Ru complex)$_1$, $Au_{25}PET_{17}$(Ru complex)$_1$, $Au_{25}PET_{18}$ and Ru complex (c).

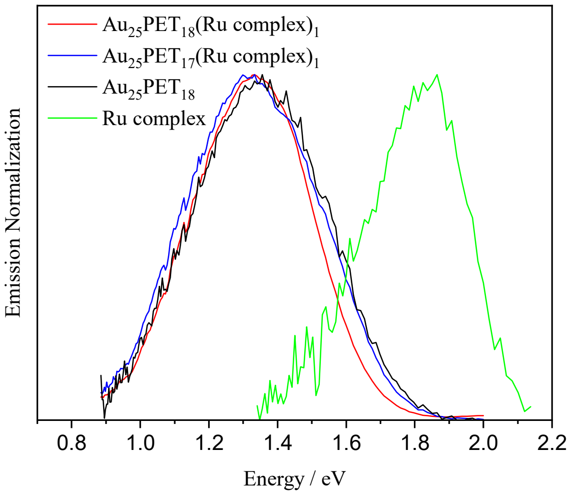

Supplementary Figure S12. Emission spectra after normalization for $Au_{25}PET_{18}$ (Ru complex)$_1$, Au PET_{17}(Ru complex)$_1$, $Au_{25}PET_{18}$ and Ru complex.

References

[1] A. Fermi, G. Bergamini, M. Roy, M. Gingras, P. Ceroni, *J. Am. Chem. Soc.* **2014**, *136*, 6395.

[2] N. Tuccitto, V. Torrisi, M. Cavazzini, T. Morotti, F. Puntoriero, S. Quici, S. Campagna, A. Licciardello, *ChemPhysChem* **2007**, *8*, 227.
